# Cuprizone and EAE mouse frontal cortex proteomics revealed proteins altered in multiple sclerosis

**DOI:** 10.1038/s41598-021-86191-5

**Published:** 2021-03-30

**Authors:** Eystein Oveland, Intakhar Ahmad, Ragnhild Reehorst Lereim, Ann Cathrine Kroksveen, Harald Barsnes, Astrid Guldbrandsen, Kjell-Morten Myhr, Lars Bø, Frode S. Berven, Stig Wergeland

**Affiliations:** 1 Proteomics Unit, Department of Biomedicine, University of Bergen (PROBE), Bergen, Norway; 2 Department of Clinical Medicine, University of Bergen, Bergen, Norway; 3 Department of Neurology, Norwegian Multiple Sclerosis Competence Centre, Haukeland University Hospital, Jonas Lies vei 65, 5021 Bergen, Norway; 4 Department of Clinical Science, University of Bergen, Bergen, Norway; 5 Computational Biology Unit, Department of Informatics, University of Bergen, Bergen, Norway; 6 Neuro-SysMed, Department of Neurology, Haukeland University Hospital, Bergen, Norway

**Keywords:** Multiple sclerosis, Cellular neuroscience, Diseases of the nervous system, Neuroimmunology, Microglia, Multiple sclerosis, Multiple sclerosis, Proteome, Proteomics, Protein-protein interaction networks

## Abstract

Two pathophysiological different experimental models for multiple sclerosis were analyzed in parallel using quantitative proteomics in attempts to discover protein alterations applicable as diagnostic-, prognostic-, or treatment targets in human disease. The cuprizone model reflects de- and remyelination in multiple sclerosis, and the experimental autoimmune encephalomyelitis (EAE, MOG1-125) immune-mediated events. The frontal cortex, peripheral to severely inflicted areas in the CNS, was dissected and analyzed. The frontal cortex had previously not been characterized by proteomics at different disease stages, and novel protein alterations involved in protecting healthy tissue and assisting repair of inflicted areas might be discovered. Using TMT-labelling and mass spectrometry, 1871 of the proteins quantified overlapped between the two experimental models, and the fold change compared to controls was verified using label-free proteomics. Few similarities in frontal cortex between the two disease models were observed when regulated proteins and signaling pathways were compared. Legumain and C1Q complement proteins were among the most upregulated proteins in cuprizone and hemopexin in the EAE model. Immunohistochemistry showed that legumain expression in post-mortem multiple sclerosis brain tissue (n = 19) was significantly higher in the center and at the edge of white matter active and chronic active lesions. Legumain was associated with increased lesion activity and might be valuable as a drug target using specific inhibitors as already suggested for Parkinson’s and Alzheimer’s disease. Cerebrospinal fluid levels of legumain, C1q and hemopexin were not significantly different between multiple sclerosis patients, other neurological diseases, or healthy controls.

## Introduction

Multiple sclerosis (MS) is a chronic immune-mediated neurological disorder characterized by chronic inflammatory demyelination, oligodendrocyte depletion, axonal loss and astrocytosis, leading to sclerotic plaques in the CNS^1^. Successful treatment relies on early diagnosis and intervention with immunomodulatory or immunosuppressive therapy. To individualize and improve treatment, better clinical methods to stratify multiple sclerosis patients into those likely to have a rapid disease progression and those with a more benign disease course are needed. Several proteomics studies on biomarkers for multiple sclerosis in body fluids have shown promising results but have not yet met the validation criteria required for clinical implementation^2,3^.

Biomarkers have been searched for in experimental disease models that reflect different MS pathophysiological mechanisms, such as the cuprizone (CPZ) model for de- and remyelination, and the T-cell mediated experimental autoimmune encephalomyelitis (EAE). Proteomics experiments have demonstrated upregulation of GFAP and downregulation of myelin proteins in CPZ c57Bl mouse brain tissue^4^. Differentially expressed proteins discovered in EAE, such as GFAP, have shown translational value when investigated in CSF from multiple sclerosis patients^5^. In contrast to MOG35-55, the full-length MOG1-125 has been shown to also induce B-cell responses. This may be of importance as B-cell depletion is among the most effective therapies in MS^6–8^. The mouse brain proteome during EAE MOG1-125 investigated in the presented study has not previously been characterized by proteomics.

Proteomic investigation of both models in parallel might identify key proteins or pathways involved in MS pathophysiology, that could be validated in brain tissue and CSF from MS patients.

## Material and methods

### Mice

Female C57Bl/6 mice obtained from Tacomic (Tornbjerg, Denmark) with a mean weight of 20.4 g ± 1.1 g were used for the experiment. The mice were housed six together in Macrolon IVC-II cages (Scanbur, Karlslunde) in standard laboratory conditions; light/dark cycles of 12/12 h, cage temperature of 22.3 ± 1 °C, relative humidity of 54 ± 5% and 75 air changes per hour. Cage maintenance was performed once weekly, and the animals were weighed twice a week by the same technician. The experiment was conducted in strict accordance with the Federation of European Laboratory Animal Science Associations recommendations, and the protocol was approved by the Norwegian Animal Research Authority (permits #2009-1767 and #2010-3814).

### Cuprizone

Demyelination was induced in eight-week-old female mice by adding CPZ (bis-cyclohexanone-oxaldihydrazone, Sigma-Aldrich, St. Louis, MO) 0.2% (w/w) to milled mouse chow for six weeks (CPZ, N = 5). The mice had ad libitum access to chow and tap water during the whole experimental period. 10 mice were randomly assigned to either CPZ exposure (N = 5) or controls (N = 5). After six weeks of CPZ exposure (CPZ-42d) the disease severity was assessed by histopathological and immunohistochemical quantification of myelin loss in the corpus callosum in mouse brain (Supplementary Fig. 1), as previously described^9^.

### Experimental autoimmune encephalomyelitis

EAE was induced in eight-week-old female mice with recombinant human MOG (rh-MOG), 1–125 from Hooke Labs, Lawrence, MA) emulsified in complete Freund’s adjuvant, injected subcutaneously at Day 0 (day of immunization). In addition, 200 ng of Pertussis toxin (Sigma-Aldrich) was injected intra-peritoneally at Day 0 and at post-immunization (p.i.) Day 2. The mice were sacrificed at the disease maximum (p.i. day 16 = EAE-16d, N = 6) and in the chronic phase (p.i. day 32 = EAE-32d, N = 12). Healthy control mice (N = 5) were sacrificed at p.i. day 32 (Supplementary Fig. 1).

### Dissection of mouse brains

The brain was excised from the mice post-mortem. The frontal cortex of the right hemisphere anterior to bregma + 1.0 mm was collected by dissection and immediately stored at – 80 ºC until further processing for proteomics analyses. The remaining part of the brain was prepared for immunohistochemistry.

### Immunohistochemistry of mouse brain tissue

The mouse brains were post-fixed in 4% formalin for at least 7 days, then paraffin embedded. All analyses were performed on 5 µm sections ± 1 mm from the bregma. The sections were stained for myelin with Luxol Fast Blue (LFB) and with hematoxylin and eosin (HE). For immunohistochemistry, sections were incubated with primary antibodies for myelin (proteolipid protein, PLP), mature oligodendrocytes (neurite outgrowth inhibitor protein A, NOGO-A) and activated microglia and macrophages (Mac-3), as described previously^10^. For each antibody, omission of the primary antibody served as negative control. Normal brain tissue from the healthy controls served as positive controls. Antibody specifications are available in Supplementary Information.

### Tissue homogenization and protein extraction

The brain tissue from frontal cortex part of the right hemisphere was thawed on ice in a 4 °C room. In triethylammonium-bicarbonate buffer containing urea, protease-, Tyr-phosphatase-, and Ser/Thr phosphatase inhibitors, the tissue was homogenized by pulse-sonication (for details, see supplementary methods). The protein concentration was measured using the BCA Protein Assay Kit in a 96 well plate (Pierce, Thermo Scientific), and the yield was approximately 1.3 mg protein per 50 mg tissue.

### TMT-labelling and protein quantification

Equal amounts of protein (urea/TEAB lysate) from the individual frontal cortex samples representing the same condition were pooled to give 100 µg protein for each condition. The samples were prepared, trypsinised and TMT-labelled as outlined in the manual for the TMTsixplex Isobaric Label Reagent Set (ThermoFisher), except that the TCEP reduction was done at RT. The peptides from the whole trypsinised samples were labelled with the TMT reagents as follows: CTR-CPZ (pool of N = 5) = TMT130, CTR-EAE (pool of N = 6) = TMT127, CPZ-42d (pool of N = 5) = TMT126, EAE-16d (pool of N = 6) = TMT128 and EAE-32d (pool of N = 6) = TMT129. The TMT labelled samples were then combined 1:1:1:1:1 into a single tube (500 µg) and dried using a vacuum concentrator (Eppendorf).

The TMT-labelled tryptic peptides were fractionated in 60 fractions using mixed-mode HPLC chromatography (Supplementary Information). Mixed-mode fractions 1–3 were pooled and fractions 7–9 were pooled, while fractions 4 to 6 containing excessive TMT reagents were excluded, resulting in a total of 53 fractions plus the unfractionated sample. The samples (0.5 µg) were subjected to LC–MS using 120 min runs with a biphasic acetonitrile gradient on a 50 cm nanoViper column (Dionex) using an Ultimate NCS-3500RS (Dionex) coupled to an LTQ-Orbitrap Velos Pro (Thermo Scientific) (Supplementary Information).

Proteome Discoverer v1.4.1.14 (Thermo Scientific) was used for identification and quantification of the TMT data. The TMT protein quantification values were then exported to Microsoft Excel and normalized within each condition (each TMT label) by log_2_-transformation of all values and subtracting the condition median. The protein quantification value (transformed to anti-log) for each protein in CPZ and EAE was divided by the respective protein quantification value in the control, control 42 days was used for the CPZ animals and control 32 days for EAE animals. The z-scores for all the protein ratios in each condition were calculated using a Gaussian probability function, and the p-values corrected using Benjamini–Hochberg as previously described^11^ (Supplemental Data 1).

### Label-free protein quantification

The individual mouse brain frontal cortex lysates from EAE-16d (N = 6), EAE-32d (N = 12), CTR-EAE (N = 5), CPZ (N = 5) and CTR-CPZ (N = 5) were trypsinised using an in-house standardized in-solution digestion protocol. The peptide samples (2.5 µg) were subjected to LC–MS on an LTQ-Orbitrap Velos Pro (Supplementary Methods).

Progenesis LC–MS v2.6 (Nonlinear Dynamics Ltd, Newcastle, UK) was used in combination with SearchGUI v1.8.9^12^ and PeptideShaker v0.17.3^13^ for label-free quantification, identification and comparison of the LC–MS proteomics data (Supplementary Methods). Progenesis LC–MS was used to calculate statistically significant up- and downregulated proteins for the label-free data based on ANOVA (p-value) with correction for multiple hypotheses testing (q-value) (Supplemental Data 2). Proteins were considered significantly regulated when the ratios over CTR were ≥ 2 and ≤ 0.7 with p < 0.05 and low q-values; q < 0.05 for CPZ-42d/CTR, q < 0.051 for EAE-16d/CTR and q < 0.11 for EAE-32d/CTR. The CTR (n = 11) was represented by values from the controls for both the CPZ (42 days, n = 6) and EAE (32 days, n = 5) experiments.

### Immunohistochemistry of human brain tissue

A total of 49 brain tissue blocks from 26 multiple sclerosis autopsy cases were obtained from the Multiple Sclerosis Biobank at the Department of Pathology, Haukeland University Hospital, Bergen. All research involving human brain tissue were conducted in accordance with the ethical standards of the regional committee for medical research ethics of Western Norway. The clinical data from the patients are summarized in Table 1. The study was approved by the regional committee for medical research ethics of Western Norway (#2013-560). Five micron thick paraffin-embedded sections were deparaffinized in xylene and rehydrated in serial aqueous dilutions of ethanol, before heat-induced epitope retrieval at pH 6.2 (Diva Decloaker antigen retrieval solution, Biocare Medical, CA, USA). To ensure optimal consistency, immunohistochemistry on all sections was performed at the same time. Primary antibodies were: anti-Legumain (LGMN), anti-PLP, anti-HLA-DR. The tissue sections were counterstained with hematoxylin and visualized by 3,3′-Diaminobenzidine (EnVision, DAKO, Glostrup, Denmark). EnVision G|2 Doublestain System (Dako), Rabbit/Mouse (DAB + / Permanent Red) was used for double staining for LGMN/HLA-DR and LGMN/GFAP to determine cellular specificity of LGMN immunopositivity.

**Table 1:** Clinical and demographic case description of brain autopsy cases.

Case #	# of tissue blocks	Post mortem delay (hrs)	MS Phenotype	Gender	Age at autopsy	Disease duration (years)	Cause of death
1	2	–	Progressive	M	34	13	Bronchopneumonia
2	1	28	Progressive	F	65	26	Congestive heart failure
4	5	–	–	M	43	–	–
5	1	8	Relapsing–remitting	F	52	20	Bronchopneumonia
7	1	24	Progressive	F	45	8	Acute pyelonephritis with sepsis
8	4	10	–	F	45	23	Bronchopneumonia
9	2	15	Progressive	M	55	36	Acute pyelonephritis with sepsis
10	2	70	Progressive	F	60	4	Bronchopneumonia
12	1	46	Progressive	M	67	42	Bronchopneumonia
13	1	13	–	M	50	20	Bronchopneumonia
14	1	29	Progressive	F	68	14	Cerebral haemorrhage
15	2	40	Progressive	M	83	–	Pseudomembraneous colitis
16	2	29	Progressive	F	49	20	Bronchopneumoina
18	3	83	Progressive	F	62	28	Bronchopneumonia
19	2	31	–	M	52	8	Acute pyelonephritis
20	3	45	Progressive	M	43	7	Bronchopneumonia
21	2	27	Relapsing–remitting	F	21	4	Bronchopneumonia
23	3	24	–	F	56	26	Bronchopneumonia
25	1	50	–	M	46	12	Suicide
26	2	23	–	F	46	–	Hyperthermia

– Information not available.

### Digitalization of stained section and characterization of multiple sclerosis lesions

PLP, HLA and LGMN stained sections were digitized in NDPI file format using a Scanscope XT slide scanner (Aperio Technologies; Vista, CA) at a resolution of 0.247 μm per pixel. White matter and cortical lesions were identified and classified by three individual investigators, according to the Bø / Trapp system^14,15^.

### Quantification of LGMN levels in brain tissue

Immunopositivity for LGMN was scored on a semi quantitative scale, based on density and stain intensity compared to LGMN immunopositivity in non-lesioned, similar tissue within the same section. Each MS-lesion was scored in the lesion center, at the lesion edge, and immediately perilesionally, avoiding other proximate lesions, as evaluated on PLP- and HLA-DR stained sections: “no difference” (0), “minor increase” (+ 1), “minor decrease” (− 1), “extensive increase” (+ 2) and “significant decrease” (− 2). Two observers evaluated all slides independently (SW and IA) and disputes were resolved by a third observer (LB). In the same area, HLA-DR-stained sections were scored from 0 to 2 for degree of microglia and macrophage reactivity: “no signs of reactive cells, only sparse ramified microglia” (0); “Signs of reactive microglia and macrophages” (1), “Presence of amoeboid, phagocytosing microglia and macrophages” (2). Correlations between LGMN immunopositivity and multiple sclerosis lesion types, and relation to degree of microglia and macrophage activation was analyzed in SPSS 24.0 in a multinomial mixed model to account for dependency in the data, with random intercept for cases and blocks.

### Human CSF samples

CSF was collected from patients that underwent diagnostic lumbar puncture at the Department of Neurology, Haukeland University Hospital, Bergen, Norway, according to the recommended consensus protocol for CSF collection and biobanking^16^. The study was approved by the regional committee for medical research ethics of Western Norway, all patients provided written informed consent, and all research involving human CSF samples were conducted in accordance with the ethical standards of the regional committee for medical research ethics of Western Norway. The included patients were 22 with RRMS, 9 without neurological symptoms, 7 with other inflammatory neurological diseases, 6 with other neurological diseases. The clinical characteristics of the cases are summarized in Table 2.

**Table 2:** Clinical and demographic case description of CSF sample cases at time of lumbar puncture.

Controls					Multiple sclerosis cases				
Diagnose at LP	EDSS	OCB status	Age (years)	Sex	Diagnose at LP	EDSS	OCB status	Age (years)	Sex
Non-neurological	N/A	Neg	74	F	RRMS	2	Pos	29	M
Non-neurological	N/A	Neg	25	F	RRMS	3	Pos	51	M
Non-neurological	N/A	Neg	36	M	RRMS	2	Pos	43	F
Non-neurological	N/A	Neg	28	M	RRMS	1.5	Pos	42	M
Non-neurological	N/A	Neg	79	M	RRMS	2	Pos	51	F
Non-neurological	N/A	Neg	63	M	RRMS	0	Pos	40	M
Non-neurological	N/A	Neg	86	F	RRMS	1.5	Pos	42	F
Non-neurological	N/A	Neg	39	F	RRMS	2	Pos	43	F
Non-neurological	N/A	Neg	68	M	RRMS	–	Pos	74	F
OIND	N/A	Neg	35	F	RRMS	2	Pos	35	F
OIND	N/A	Pos	32	M	RRMS	1.5	Pos	38	F
OIND	N/A	Pos	45	F	RRMS	3	Pos	35	F
OIND	N/A	Neg	63	F	RRMS	1.5	Pos	42	M
OIND	N/A	Neg	33	M	RRMS	1.5	Pos	29	F
OIND	N/A	Neg	35	M	RRMS	1	Pos	28	F
OIND	N/A	Neg	35	M	RRMS	1	Pos	33	F
OND	N/A	Neg	50	F	RRMS	1	Pos	29	F
OND	N/A	Neg	28	F	RRMS	2	Pos	32	F
OND	N/A	Neg	36	F	RRMS	1	Pos	37	F
OND	N/A	Neg	38	F	RRMS	1	Pos	35	F
OND	N/A	Neg	37	F	RRMS	0	Pos	28	F
OND	N/A	Neg	33	M	RRMS	1	Pos	41	F

*LP* lumbar puncture, *EDSS* expanded disability status scale, *OCB* oligoclonal bands, *CSF* cerebrospinal fluid. – information unavailable.

### Parallel reaction monitoring to quantify LGMN, C1Q and HEMO in CSF

The concentration of the candidate protein LGMN, C1Q and HEMO in CSF from multiple sclerosis (MS), other neurological disease (OND) controls and healthy controls (NN) were determined using parallel reaction monitoring (PRM) both in absolute and relative terms.

The absolute amounts of these proteins were measured. All CSF samples were in-solution digested as previously described. About 1.5 µg CSF digested protein were injected for Legumain and C1q and about 0.2 µg for Hemopexin. Relative PRM was assisted by stable isotope-labeled internal standard (SIS) peptides (MS vs OND). The unique LGMN peptide DYTGEDVTPQNFLAVLR (a.a. 101–117), HEMO NFPSPVDAAFR and C1Q FQSVFTVTR were chosen to cover the protein sequence toward both termini (Supplementary Data 3, Supplementary Information).

### Proteomics data post-processing and availability

Perseus v1.4.1.3^17^ was used to generate the unsupervised clustering heatmap with dendrograms of z-score normalized data using the default settings. GraphPad Prism 6 (GraphPad Prism Software) was used for the statistical analyses and graphics and Venn diagrams were created using Venny (http://bioinfogp.cnb.csic.es/tools/venny/index.html). Ingenuity Pathway Analysis (IPA, Ingenuity Systems, http://www.ingenuity.com) was used for pathway and function analyses. DAVID^18^ was used to investigate the regulated proteins in KEGG pathways^19^. MADGENE^20^ was used to convert the mouse accession numbers to human orthologues for comparison against the results in CSF-PR^21^. Additional information on the tools used can be found in Supplementary Information.

The mouse discovery proteomics TMT and label-free data have been deposited to the ProteomeXchange Consortium^22^ via the PRIDE partner repository^23^ with the dataset identifier PXD002318. An overview of the raw data files and the analyzed data are available (Supplementary Data 1–2).

## Results

The proteomic analysis uncovered differentially regulated proteins in the frontal cortex part of EAE and CPZ mice of which human orthologues were investigated as disease markers for multiple sclerosis.

### Proteins regulated in CPZ and EAE frontal cortex and human orthologues in CSF

**Figure 1:**
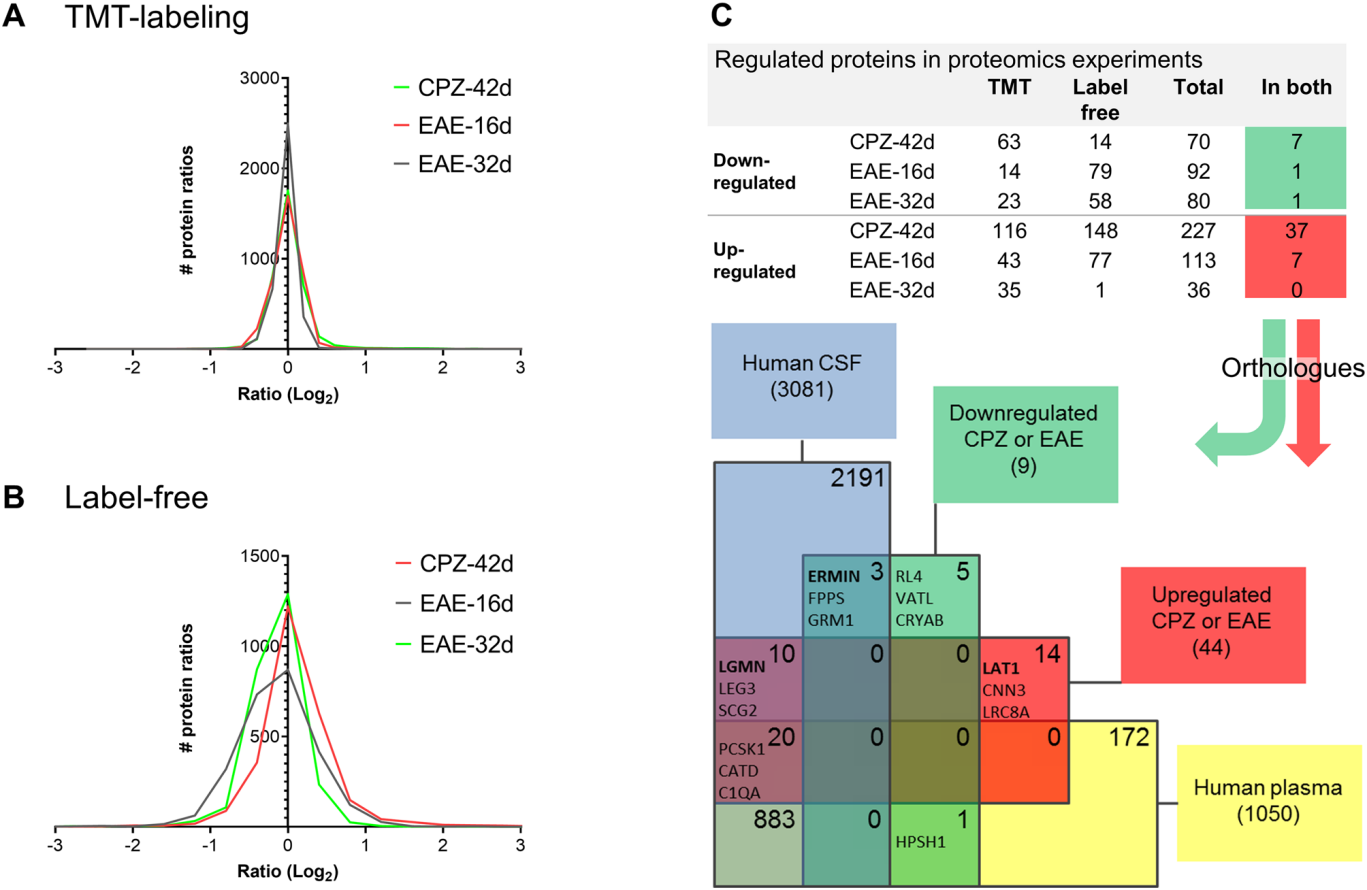
Proteins quantified in EAE and CPZ frontal cortex and human orthologues in CSF and plasma. (**A**) Frequency distribution of all normalized log_2_ ratios for proteins quantified using TMT-labeling in frontal cortex in CPZ-42d, EAE-16d and EAE-32d divided by the respective controls. The TMT data was normalized using the log_2_ minus the condition median value. The relative numbers of proteins in the ratio bins were automatically determined by GraphPad and presented as continuous lines. (**B**) Frequency distribution of all normalized log_2_ ratios for proteins quantified using label-free in frontal cortex in CPZ-42d, EAE-16d and EAE-32d divided by the respective controls. The label-free data was normalized using the default algorithm in Progenesis LC–MS. The relative numbers of proteins in the ratio bins were automatically determined by GraphPad and presented as continuous lines. Slight shifts/shoulders in the frequency distribution plots are due to that the label-free protein quantification is based on the sum of the quantified peptide features with proteins ID’s assigned and the peptides being unique for the protein. The normalization was performed on all peptides. The log2 protein ratios are thus based on a selection of the peptide features from the LC–MS runs. (**C**) Number of significantly regulated proteins in the conditions CPZ-42d, EAE-16d and EAE-32d relative to respective controls using TMT-labelling and label-free proteomics. The numbers of proteins regulated in both methods are shown, downregulated squared in green and upregulated in red. The human orthologues of the proteins regulated in both TMT and label-free were compared to the CSF and plasma proteins in the CSF-PR database^21^. The Venn diagram shows how many protein accession numbers that were shared by the respective proteomes, and some of the most relevant regulated proteins.

The cuprizone mice were sacrificed at the demyelination/re-myelination stage (CPZ-42d), and the EAE mice at the disease peak (EAE-16d) and at the recovery phase (EAE-32d) (Supplementary Fig. 1). The number of proteins (unique protein accession numbers and protein groups) quantified in CPZ and EAE mouse brains with respective controls was 3664 in the TMT-labelling experiment (Supplementary Data 1) and 2582 in the label-free experiment (Supplementary Data 2), a total of 4375 proteins. The distributions of protein ratios of CPZ-42d, EAE-16d or EAE-32d relative to control are presented in Fig. 1A,B.

The proteins significantly regulated in the TMT experiment and verified as regulated in the label-free experiment were considered to be the most certain changes (Table 3). Of these, the proteins with human orthologues previously detected in human CSF and/or plasma were identified using the CSF-PR database^21^ (Fig. 1C, Table 3). These protein candidates were considered especially interesting as prognostic- and diagnostic markers.

**Table 3:** Significantly regulated proteins in both TMT-labeling and label-free.

Accession	Protein		TMT-labeling																		Label-free											
			P		S		CPZ-42d				EAE-16d				EAE-32d						CPZ-42d		EAE-16d				EAE-32d					
							FC		q		FC		q		FC		q		P		FC	q	FC		q		FC		q	hCSF		hP
**(A) Upregulated in CPZ-42d**																																
P14106	C1QB		5		19		5.3		***		0.8				0.8				2		19.4	*’	1.5				1.3			Y		Y
Q02105	C1QC		6		14		5.3		***		0.8				0.8				3		9.1	*’	1.2				1.3			Y		Y
O89017	LGMN		6		11		4.0		***		0.7				0.7				1		64.9	*	1.9				1.3			Y		
P98086	C1QA		6		20		3.8		***		0.9				0.9				4		6.8	*’	1.1				1.1			Y		Y
P16110	LEG3		5		5		3.7		***		1.1				0.8				2		338.7	*’	2.3				1.1			Y		
Q99L04	DHRS1		15		42		3.7		***		0.8				0.8				3		4.0	*’	0.9				0.9					
P24452	CAPG		6		9		3.4		***		0.9				0.9				1		4.7	*’	1.2				1.0			Y		
P20152	VIME		30		540		3.1		***		1.2				1.1				22		7.7	*’	1.6				1.2			Y		Y
P20060	HEXB		18		48		2.9		***		1.0				1.0				6		5.0	*’	1.6				1.2			Y		Y
P03995	GFAP		38		658		2.6		***		1.3				1.1				18		7.2	*’	1.5				1.0			Y		
P11835	ITB2		5		5		2.6		***		0.8				0.8				1		8.2	*’	0.5				0.7			Y		
Q9Z127	LAT1		3		7		2.4		***		0.5		***		0.6		***		1		4.5	*’	0.5				0.6					
P26041	MOES		20		46		2.3		***		1.1				1.0				4		2.6	*’	0.9				0.9			Y		Y
O35639	ANXA3		15		25		2.2		***		1.0				1.0				1		5.9	*’	1.4				1.3			Y		
P18242	CATD		16		119		2.2		***		1.0				0.9				10		2.8	*’	1.3				1.0			Y		Y
Q9DAW9	CNN3		8		22		2.1		***		1.0				1.0				4		4.0	*’	1.2				1.0					
Q9D379	HYEP		8		11		2.0		***		0.8				0.8				2		2.9	*’	0.6				0.7					
P10605	CATB		9		57		1.9		***		0.9				1.0				7		3.4	*’	1.0				0.9			Y		Y
Q8BTM8	FLNA		30		40		1.9		***		0.8				0.9				2		1.5	*’	1.4				1.0			Y		Y
P60824	CIRBP		4		17		1.9		***		1.0				1.0				1		4.8	*’	1.2				1.1					
Q9DCJ9	NPL		7		11		1.8		***		1.0				1.0				1		19.8	*’	1.4				0.7					
Q9WVA4	TAGL2		11		38		1.8		***		1.1				1.0				3		2.9	*’	1.2				0.9			Y		Y
P09055	ITB1		4		5		1.7		***		0.9				0.8				1		2.8	*	0.4				0.6			Y		Y
P10852	4F2		20		107		1.6		**		0.8				0.8				12		1.9	*’	0.8				0.8			Y		Y
Q80WG5	LRC8A		4		4		1.6		**		0.8				0.8				1		2.1	*’	0.8				0.7		§			
P16045	LEG1		6		25		1.6		**		0.9				0.9				2		3.1	*’	1.0				1.0			Y		
P61620	S61A1		2		4		1.6		**		0.8				0.8				1		2.5	*	0.4		*		0.6					
Q9CQI6	COTL1		13		138		1.5		*’		1.0				1.0				5		2.1	*’	0.9				0.9			Y		
P16675	PPGB		3		4		1.5		*’		0.9				1.0				1		3.2	*’	0.9				1.0			Y		Y
P07356	ANXA2		11		20		1.5		*		1.0				1.0				3		2.7	*’	0.7				0.7			Y		Y
Q9DCD0	6PGD		15		27		1.5		*		0.9				0.9				5		1.7	*	0.8				0.8			Y		Y
Q9Z110	P5CS		14		22		1.5		*		0.9				1.0				2		2.9	*’	0.8				0.9					
O89086	RBM3		5		19		1.5		*		1.0				1.0				1		5.0	*’	1.3				1.1					
P51880	FABP7		4		20		1.5		*		0.9				1.4		***		6		2.9	*’	1.1				1.9			Y		
Q8CGC7	SYEP		29		34		1.4		*		0.8				0.8				3		2.1	*’	0.6				0.7					
P08030	APT		6		11		1.4		*		1.1				1.0				1		2.2	*	1.1				1.0					
Q8VDD5	MYH9		47		94		1.4		*		0.9				0.9				8		1.8	*’	0.8				0.8			Y		Y
**(B) Downregulated in CPZ-42d**																																
Q5EBJ4	ERMIN		7		21		0.5		***		1.3				1.1				3		0.5	*’	0.9				0.8			Y		
P47911	RL6		13		25		0.5		***		1.3				1.0				1		0.5	*’	0.9				0.8					
P23927	CRYAB		6		67		0.6		***		1.2				1.0				4		0.5	*	1.2				1.0					
Q9D8E6	RL4		13		29		0.7		*’		1.1				1.0				1		0.5	*	0.6				0.8					
Q61699	HS105		36		78		0.7		*		1.0				1.0				12		0.6	*’	0.9				0.9					Y
Q920E5	FPPS		9		14		0.7		*		1.0				1.0				2		0.6	*	0.7				0.8			Y		
P14148	RL7		6		10		0.7		*		0.9				0.9				1		0.6	*	0.7				0.8					
**(C) Upregulated in EAE-16d**																																
Q91X72	HEMO		15		28		0.9				1.8		***		1.2				3		0.9		3.3		*		1.5			Y		Y
P07758	A1AT1		4		8		1.0				1.7		***		1.2				1		0.9		4.4		*		1.8			Y		Y
Q9QXV0	PCSK1		8		102		1.2				1.5		**		1.1				4		2.1		2.2		*		1.1			Y		Y
Q8K019	BCLF1		7		7		1.1				1.4		*’		1.4		***		1		1.3		1.4		*		1.2					
Q03517	SCG2		28		160		1.1				1.4		*		1.1				10		1.5		2.6		*		1.5			Y		
P22005	PENK		7		35		1.1				1.4		*		1.2				1		0.7		6.0		*		2.6			Y		Y
Q9JJF0	NP1L5		5		10		0.9				1.4		*		1.1				1		1.4		2.2		*		1.0					
**Mouse**																																
**(D) Downregulated in EAE-32d**																																
P97772	GRM1		4		4		1.1				0.7				0.7		*		2.0		1.8		0.5				0.5		§	Y		

(A) Proteins upregulated in CPZ-42d in both TMT and label-free. (B) Proteins downregulated in CPZ-42d in both TMT and label-free. (C) Proteins upregulated in EAE-16d in both TMT and label-free. (D) Protein downregulated in EAE-32d in both TMT and label-free. For all tables: quantified proteins, number of unique peptides (P) and spectra (S) with fold change relative to respective controls (FC) are shown for CPZ-42d, EAE-16d and EAE-32d. The p-values for the regulated proteins were corrected for multiple hypothesis testing to control false discovery rates, resulting in adjusted p-values (q-values). The q-values are denoted in the table as follows: §, q < 0.1; *, q < 0.05; *’ q < 0.01; **, q < 0.005, *** q < 0.0005. For the TMT-data, the z-scores for all the protein ratios in each condition were calculated using a Gaussian probability function, and the p-values corrected using Benjamini–Hochberg (q-values), as previously described^11^. For the label-free data Progenesis LC–MS was used to calculate statistically up- and downregulated proteins based on ANOVA (p-value) with correction for multiple hypotheses testing based on FDR (q-value). Consistent regulation in all individuals in CPZ-42d allowed a stringent limit for the corrected p-value from multiple hypothesis testing (q < 0.05), a moderate in in EAE-16d (q < 0.051) and a higher limit in EAE-32d (q ≤ 0.1). Human orthologues present in human cerebrospinal fluid (hCSF) and plasma (hP) according to the CSF-PR database^21^ are indicated with yes (Y = yes).

In CPZ-42d several significantly regulated proteins, of which human orthologues were found using CSF-PR, were associated with inflammation and protease activity (upregulation of LGMN, LEG1, LEG3), migration and integrin signaling (upregulation of ITB1, ITB2, FABP7), microglia/macrophage signaling (upregulation of C1Q, CAPG, HEXB), astrocytosis (upregulation of GFAP, VIME) and demyelination (downregulation of ERMN) (Table 3A,B). ERMN, involved in myelinogenesis and oligodendroglia maturation^24^, was the most downregulated protein in CPZ-42d and the human orthologue was found in CSF-PR.

The proteins regulated in EAE-16d indicate that the processes of inflammation, microglia activation, astrocytosis and demyelination were less prominent compared to CPZ-42d, and even less in EAE-32d than in EAE-16d. In EAE-16d the acute phase associated proteins HEMO and A1AT1 and the granin family proteins PCSK1 and SCG2 were significantly increased (Table 3C). In addition, proteins involved in glutamate homeostasis were regulated in EAE-16d (upregulation of PENK), and in EAE-32d (downregulation of GRM1) (Table 3D).

### Pathway analyses of the quantified proteins

**Figure 2:**
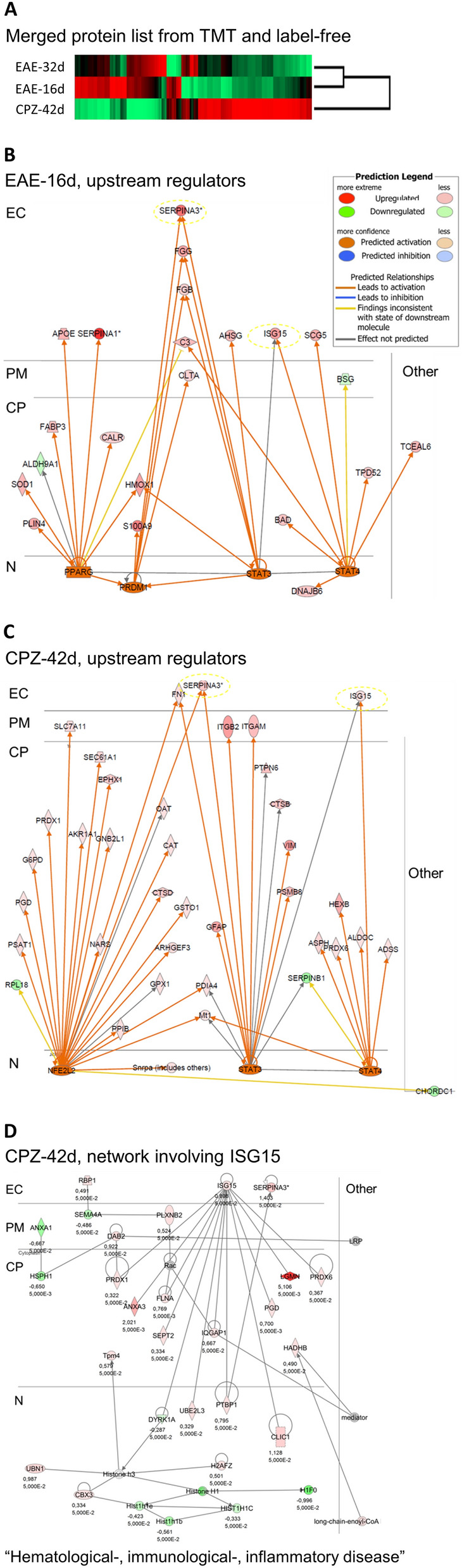
Clustering and pathway analyses of proteins quantified in EAE and CPZ. The protein ratios relative to respective controls for EAE-16d, EAE-32d and CPZ-42d from TMT and label-free were combined prior to analyses in Perseus and IPA. (**A**) Unsupervised hierarchical clustering of the TMT and label-free protein ratio list (Perseus). (**B,C**) The significance level of regulation (IPA “p-value”) was set to 0.005 if the protein was significantly regulated with more than 1.2-fold in both TMT and label-free experiments and 0.05 if only in one experiment. Predicted activated upstream regulators, such as STAT3 and STAT4, are illustrated as networks together with associated proteins from our uploaded dataset EAE-16d (**B**) and CPZ-42d (**C**). Predicted network in IPA for CPZ-42d “Hematological disease, Immunological disease, Inflammatory disease” involving ISG15 (upregulated in CPZ-42d, in EAE-16d and EAE-32d only in TMT), SERPINA3 (upregulated in CPZ-42d only in TMT), and LGMN (highly upregulated in CPZ-42d) (**D**). The average log_2_ ratio and the “IPA p-value” from the combined TMT and label-free protein list are shown for each protein node. The proteins significantly altered with a fold change higher than 20% in both TMT and label-free were considered more significant (IPA p-value 0.005) than the proteins regulated in only one of the experiments (IPA p-value 0.05). The IPA prediction legend is shown as an insert in (**B**). *EC* extracellular space, *PM* plasma membrane, *CP* cytoplasm, *N* nucleus.

The data from the TMT experiment and the label-free experiment were combined and analyzed by unsupervised clustering. As expected, the EAE-16d and EAE-32d datasets appeared more similar to each other than to CPZ-42d (Fig. 2A). The combined data were analyzed using Ingenuity Pathway Analysis (IPA), and upstream regulators were predicted based on the protein regulations in EAE-16d and CPZ-42d (Fig. 2B,C). An activation of STAT3 (upregulated in CPZ-42d in TMT) and of STAT4 (not detected in our datasets) were predicted from both the EAE-16d and the CPZ-42d datasets (Fig. 2B,C), and activation of ISG15 was common for both STATs. ISG15 was upregulated in CPZ-42d, in EAE-16d and EAE-32d but only in TMT. The STATs have previously been discussed as possible treatment targets in multiple sclerosis^25^. The predicted networks with the highest scores in IPA are presented in Supplementary Fig. 2.

### Investigation of regulated proteins in multiple sclerosis

One of the most upregulated proteins in CPZ-32d was legumain (LGMN), and the human orthologue has previously been detected in human CSF (CSF-PR). LGMN was one of the 19 regulated proteins that contributed to significant overrepresentation of the lysosomal KEGG pathway (FDR = 7 × 10^-7^) when all regulated proteins were analyzed in DAVID^18^. In the lysosomes, LGMN is involved in processing proteins for MHC class II antigen presentation^26^, a biological function relevant to multiple sclerosis pathology. ISG15 in the predicted STATs networks (Fig. 2B,C) has been reported to have protein interaction with LGMN^27^ as illustrated in one of the predicted networks for CPZ-42D (Fig. 2D). LGMN has previously been shown to be expressed on macrophages in active demyelinating white matter lesions^28^, and was further investigated in this study in both white and grey matter using IHC, and in human CSF using PRM.

C1Q proteins, important in complement activation in multiple sclerosis have previously been investigated in multiple sclerosis using IHC^29^, and were among the most upregulated proteins in CPZ-42d. C1Q proteins were investigated using parallel reaction monitoring (PRM) in CSF from multiple sclerosis patients.

HEMO, the most upregulated protein in EAE (Table 3C, Supplementary Fig. 2B), has previously been suggested as affected candidates from proteomics of CSF from rats with EAE^30^ and was upregulated in plasma of pediatric multiple sclerosis patients^31^. HEMO was investigated further in human CSF using PRM.

### Legumain expression in multiple sclerosis lesions

**Figure 3:**
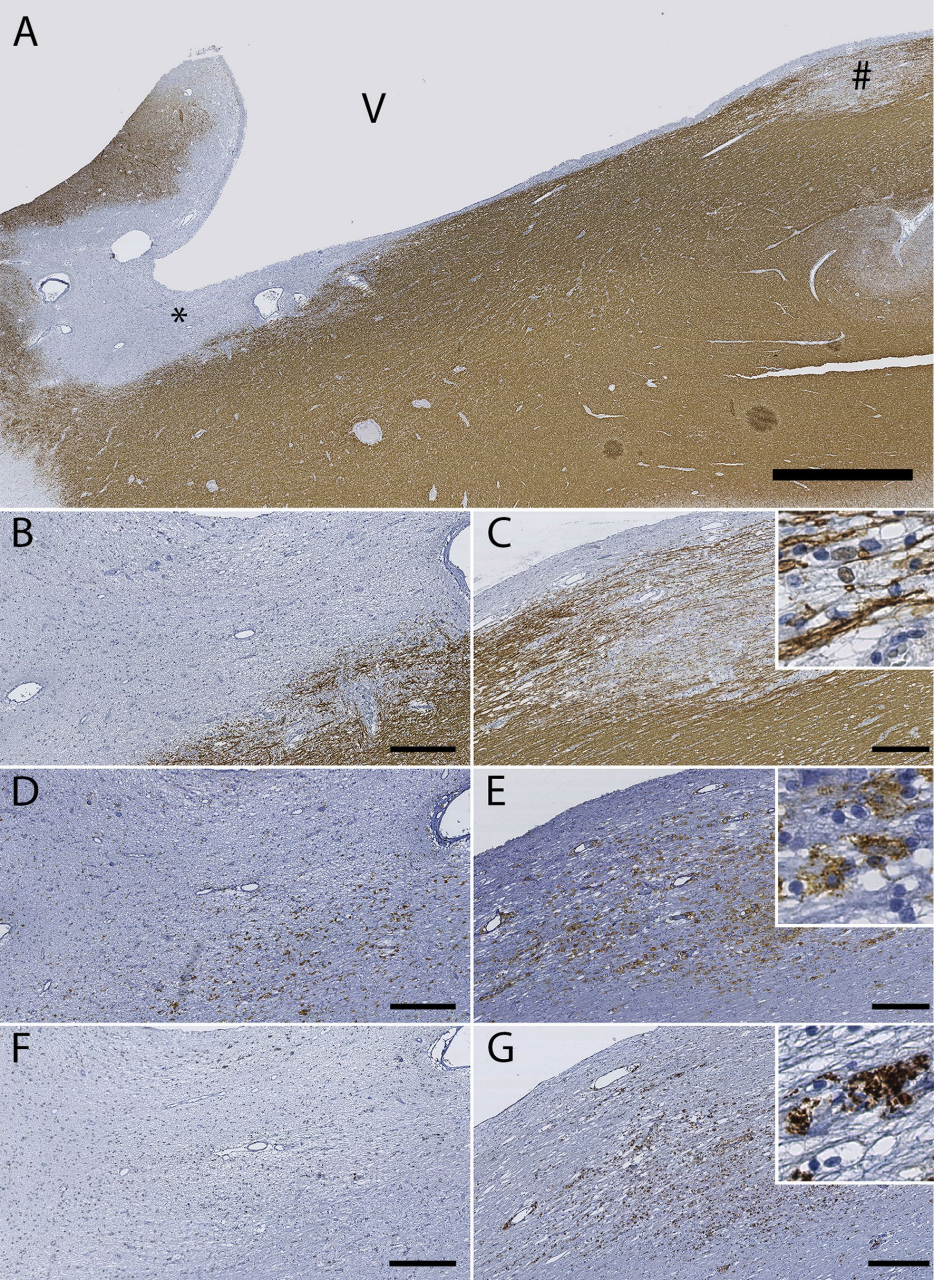
Expression of LGMN in white matter lesions. (**A**) Myelin (PLP) immunostained section with an active lesion (# in **A**, images **C,E,G**) and an inactive lesion (* in **A**, images **B,D,F**). Scale bar 2 mm. (**B,C)** Myelin (PLP) immunostaining of lesions indicated in **(A)** at higher magnification. Scale bar 300 μm. Insert in **(C)** shows intracellular PLP-positive myelin debris, suggesting an early active lesion with ongoing demyelination. (**D,E**) HLA-DR immunostaining of lesions indicated in **(A)**. Scale bar 300 µm. Insert in **(E)** shows activated HLA-DR positive cells. (**F,G**) LGMN immunostaining of the lesions indicated in **(A)**. Scale bar 300 µm. In **(G)**, increased LGMN expression is seen throughout the active lesion, while in the chronic inactive lesion **(F)**, there is reduced LGMN expression, due to general hypocellularity and absence of HLA-DR positive cells. Insert in **(G)** shows a representative image of LGMN immunostaining in a pattern compatible with cytoplasmic lysosomes.

**Figure 4:**
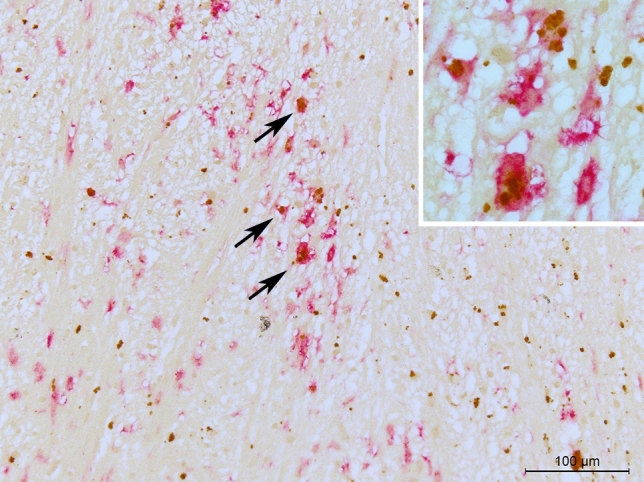
Double staining of lesion for identification of LGMN positive cells. (LGMN (DAB, brown) in HLA-DR (Alkaline phosphatase, reddish pink) positive cells (arrows),  × 20 magnification and insert at  × 63 magnification.

**Figure 5:**
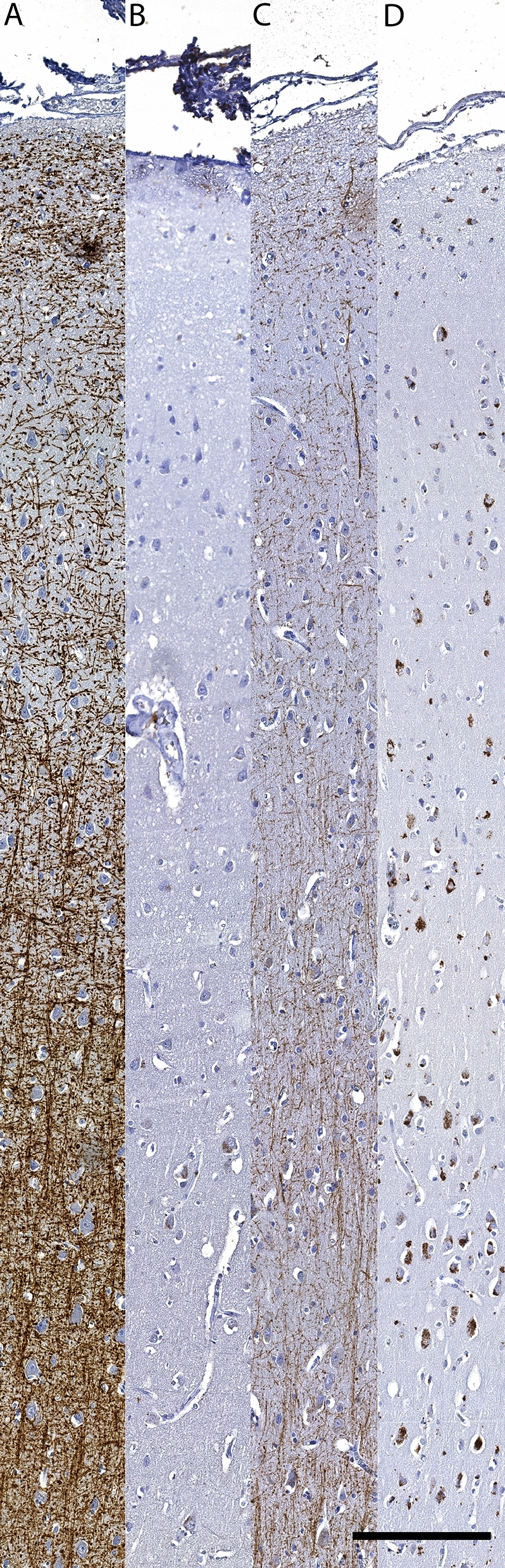
Increased LGMN expression in multiple sclerosis normal appearing cerebral cortex. LGMN (**A,B**) and myelin **(C,D**) immunostaining of cortex from a multiple sclerosis case (**B,D**) and control (**A,C**). Throughout all cortical layers there is reduced myelin content, and an increase in LGMN immunopositivity. Scale bar 200 µm.

LGMN levels were investigated in human brain tissue autopsy samples using IHC, and expression means and precision estimates in white matter and cortical lesions are given in Table 4. LGMN immunopositivity was significantly increased in the lesion center and at the lesion edge of active and chronic active white matter lesions compared to inactive lesions (Table 5, Fig. 3). There was no significant difference of LGMN immunopositivity in cortical lesions, compared to normal-appearing gray matter. There was a significant association between LGMN immunoreactivity in white matter MS lesion areas and density of HLA-DR immunopositive cells infiltration, where increased LGMN expression in the lesion center and edge were associated with the presence of HLA-DR positive cells with the morphology of foamy macrophages/activated microglia (Table 5, Fig. 3). Double labelling for HLA-DR / LGMN, and GFAP / LGMN showed predominantly colocalization of LGMN with HLA-DR-immunopositive, and not GFAP- immunopositive cells (astrocytes) (Fig. 4). A subpopulation of cortical neurons was weakly legumain immunopositive. This was not observed in controls, but the available tissue did not allow for comparison of neurons from the same anatomical area and layers. Brain autopsy samples from three control cases without known neurological diseases were available for PLP, HLA-DR and LGMN staining, and by visual comparison, the overall LGMN expression was higher in the cortex of multiple sclerosis cases compared to the controls (Fig. 5).

**Table 4:** LGMN scores in human brain tissue samples.

Lesion sublocation	White matter lesions							Cortical lesions							
	Active		Chronic active		Inactive			Type 1		Type 2		Type 3		Type 4	
	M	SD	M	SD	M	SD		M	SD	M	SD	M	SD	M	SD
Center	1.6	0.7	0.8	0.9	0.0	0.9		0.6	1.0	-0.1	0.3	0.0	0.8	0.3	0.5
Edge	1.5	0.5	1.1	0.7	0.4	0.8		0.7	1.0	0.3	0.5	0.1	0.8	0.1	0.4
Perilesionally	0.3	0.5	0.3	0.6	0.0	0.3		0.1	0.7	0.2	0.4	0.0	0.7	0.0	0.0

*M* mean, *SD* standard deviation, Type 1 leucocortical, Type 2 subpial, Type 3 intracortical, Type 4 throughout cortex.

**Table 5:** Multinominal linear regression analyses of LGMN score by lesion type and HLA-DR.

	N	%	Center			Edge			Peri-lesion		
			B	95%CI	p	B	95%CI	p	B	95%CI	p
**White matter lesions**											
LR χ^*2*^			F_df 2_ = 23.5, p < 0.001			F_df 2_ = 14.9, p = 0.001			F_df 2,48_ = 8.8, p = 0.001		
Active	8	13.3%	4.00	[2.22,6.22]	** < 0.001**	2.70	[1.17,4.46]	**0.001**	1.60	[−0.53,3.87]	0.129
Chronic active	17	28.3%	1.60	[0.45,2.86]	**0.008**	1.60	[0.41,2.91]	**0.011**	1.50	[−0.29,3.64]	0.108
Chronic inactive*	35	58.3%	0*	–	–	0*	–	–	0*	–	–
**Gray matter lesions**											
LR χ2			F_df 3_ = 7.6, p = 0.056			F_df 3_ = 3.7, p = 0.154			F_df 3_ = 1.6, p = 0.662		
Type 1—leucocortical	11	17.7%	1.29	[−0.86,3.56]	0.248	1.74	[−0.45,4.05]	0.126	0.00	[−3.26,3.26]	< 1
Type 2—intracortical	16	25.8%	-1.47	[−3.42,0.41]	0.129	0.52	[−1.35,2.45]	0.593	1.39	[−1.21,4.31]	0.322
Type 3—subpial	28	45.2%	-0.90	[−2.56,0.75]	0.283	-0.01	[−1.67,1.72]	0.990	0.38	[−2.06,3.02]	0.770
Type 4—throughout cortex*	7	11.3%	0*	–	–	0*	–	–	0*	–	–
**HLA-DR**											
LR χ^*2*^			F_df 2_ = 35.7, p < 0.001			F_df 2_ = 41.7, p < 0.001			F_df 2_ = 8.0, p = 0.018		
Inactive	74	66.1%	0*	–	–	0*	–	–	0*	–	–
Reactive	29	25.9%	2.28	[1.35,3.28]	** < 0.001**	2.64	[1.67,3.7]	** < 0.001**	1.39	[0.07,2.83]	0.064
Active, amoeboid	9	8.0%	3.27	[1.83,4.8]	** < 0.001**	3.22	[1.82,4.71]	** < 0.001**	2.18	[0.43,3.91]	**0.009**

*B* coefficient, *CI* confidence interval, * reference category, *LR χ2* loglinear chi square, *Df* degrees of freedom.

Significant p-values after sequential Bonferroni correction indicated in bold typeface.

### Quantification of upregulated proteins in CSF from multiple sclerosis patients

In order to investigate whether the levels of LGMN, C1Q (upregulated in CPZ) and HEMO (upregulated in EAE16d) were significantly affected in CSF samples from multiple sclerosis patients (Table 2), a targeted LC–MS/MS PRM assay for quantification was developed. The protein levels for the three candidates were not significantly different between relapsing–remitting multiple sclerosis patients (n = 22) and OIND (n = 7) OND (n = 6) or healthy controls (n = 9) as illustrated in Supplementary Data 3. These results demonstrate that LGMN, C1Q and HEMO did not show potential as a diagnostic- or prognostic marker in the samples investigated.

## Discussion

A high number of identified and regulated proteins in the brains of mice subjected to either EAE or CPZ exposure allowed for an extensive comparison of the brain proteomes in these two models. Several differences, both at single-protein and protein network levels were found. Proteome comparison between CPZ-42d and controls indicate that pathways annotated to inflammation, migration and integrin signaling, microglia/macrophage activation, astrocytosis and demyelination were affected during CPZ de-/remyelination. These events were less prominent in the brains of EAE mice at the disease peak (EAE-16d), and even less at the recovery phase (EAE-32d). Acute phase associated proteins, granins and glutamate homeostasis proteins were significantly altered in EAE-16d, and the latter were also altered in EAE-32d. Although inflammation and T-cell infiltration predominantly occurs in the spinal cord in the EAE model^32^ pathological changes also occur in the brain ^33,34^. In the latter studies, the areas outside the inflammatory foci revealed regulation of proteins potentially involved in neurodegenerative processes rather than regulation of proteins in general inflammatory processes. This supports the fact that we did not observe many proteins involved in inflammation in the EAE mouse brains.

LGMN was highly upregulated in CPZ-42d (Table 3A) and associated with protein networks known to be affected by multiple sclerosis (Fig. 2). LGMN is a lysosomal multifunctional protein that can exert situation dependent endopeptidase, carboxypeptidase and ligase activity^26^. LGMN has been shown to bind to ISG15 as illustrated in the predicted ISG15 network (Fig. 2D), and might thus be a target substrate for ISGylation to regulate LGMN activity. ISGylation is the conjugation of ISG15 to target substrates with the help of an enzymatic cascade, suggested to play a role as an endogenous neuroprotective mechanism^35^. Expression of ISG15 and its conjugation is most likely the by-product of IFN 1 response. ISG15 acts as a chemotactic factor, and the secreted form can induce natural killer cell proliferation.

In addition to LGMN, four other lysosomal proteases were upregulated in CPZ-42d, the cathepsins CATB, CATD, CATS and CATZ, and the lysosomal pathway was overrepresented in this dataset according to the DAVID analysis. An increased activity of cathepsins might contribute to increased multiple sclerosis disease activity^36^. LGMN has previously been suggested to play a role in antigen presentation in multiple sclerosis^37^.

The increased LGMN expression in HLA-DR (MHC-II) immunopositive cells indicate an association with inflammatory activity in the MS-lesion^38^. Although we were not able to confirm increased immunopositivity for LGMN in neurons in normal-appearing cortex of MS patients due to limitations within the tissue samples, translocation of LGMN from the lysosomes to cytosol has been observed in other pathological conditions such as Alzheimer’s disease^39,40^ and colorectal cancer^41,42^. In microscopically normal appearing multiple sclerosis brain tissue, the LGMN gene, among other genes related to proteolytic processing, is hypo-methylated and more extensively transcribed compared to controls without neurological disease^43^.

The network “Cellular assembly and organization, Neurological disease, Organismal development” was predicted with a high score for EAE-16d in IPA (Supplementary Fig. 2B) and involved the acute phase proteins HEMO (hemopexin, gene: HPX), A1AT (alpha-1-antitrypsin 1–1, gene: SERPINA1) and CO3 (complement 3, gene: C3). HEMO and A1AT1 were highly upregulated in EAE-16d (Table 3C) and have previously been suggested as affected candidates from proteomics of CSF from rats with EAE^30^. HEMO has also been observed to be upregulated in plasma of pediatric multiple sclerosis patients^31^, however, the protein was not significantly regulated in CSF in our PRM study.

When looking at other serpin family members in our dataset, we found that SPA3K, ANT3 and AIAT2 also were upregulated in EAE-16d suggesting a role in multiple sclerosis pathophysiology for several of the serpin family members. SPA3N (Alpha-1-antichymotrypsin), a verified candidate biomarker in CSF for secondary progressive multiple sclerosis^44^ was upregulated in CPZ42d (only found in TMT). In contrast to EAE-16d, where only upregulation of CO3 was observed, complement C1Q subunits A, B and C were highly upregulated in CPZ-42d (Table 3A). This observation is in accordance with the high degree of demyelination and microglia activity seen in CPZ-42d (Supplementary Fig. 1C). In multiple sclerosis, activation of the classical complement pathway has been shown to occur especially in reactive astrocytes adjacent to clusters of microglia with opsonized myelin and damaged axons^29^.

An increase of immunoglobulin IGKC was observed in both EAE-16d and EAE-32d but not in CPZ-42d and an increase has been reported to occur in CSF from multiple sclerosis patients^45^. The increase in EAE, and not the CPZ model, could be due to the involvement of T-cell infiltration in EAE.

In our frontal cortex proteomics approach, we observed that most of the proteins regulated in CPZ were not regulated in EAE and vice versa. As we were analyzing brain tissue form the frontal hemisphere, we would expect to characterize proteins from both glia cells and neurons given that the glia to neuron ratio in this tissue is suggested to be approximately 1:1^46^. However, in EAE and CPZ mice the numbers of certain glia cells and neurons are likely to have been altered ^10,47^ thereby affecting the global brain proteomes investigated. We did not observe differential regulation in neuronal markers in EAE or CPZ, however an increased number of astrocyte protein markers (increased GFAP and VIME) and a decreased level of oligodendrocyte protein markers (MOG, MAG, MBP, CLD11 and ERMIN) was observed in CPZ-42d with only tendencies in EAE-16d and EAE-32d. ERMIN, which was significantly downregulated in CPZ-42d (Table 3B) is an oligodendrocyte specific cytoskeleton-related protein involved in myelination in humans^48^. ERMIN has not previously been associated with multiple sclerosis and the regulation of this protein should thus be investigated further during phases of de- and remyelination in patients.

In CPZ-42d we observed upregulated macrophage/microglia markers ITAM (CD11B) and HEXB^49^ indicating an increase in the number of these cell types; this was not observed in EAE. Furthermore, the galectins LEG3 (LGALS3) and LEG1 (LGALS1) were upregulated in CPZ-42d (fold change 3.7 and 1.6, respectively), and associated with the “Tissue development” network in IPA (Supplementary Fig. 2C). In cuprizone mice, it has been demonstrated that LEG3 is expressed in microglial cells, where one of its roles is to facilitate the onset of remyelination^50^. These findings are in accordance with our previous observations of astrocytosis, increased macrophage/microglia and loss of oligodendrocytes in the CPZ model^10^. In EAE, lesions are predominantly observed in the spinal cord^32^, however, pathological changes have also been seen in brain tissue remote from inflammatory areas^33,34^, as demonstrated in this study. Thus, protein regulations we observed in EAE might be pathological events in areas adjacent to inflammatory lesions, which may potentially reflect changes at an early stage prior to lesion formation, being involved in protecting healthy tissue and assisting repair of inflicted areas.

## Conclusion

Based on the regulation of proteins quantified in the frontal cortex different pathway- and biological function signatures could be assigned to cuprizone and EAE models of multiple sclerosis. Differences in processes relevant to multiple sclerosis pathophysiology were indicated, such as myelinogenesis, lysosomal pathways, amino acid transport, acute phase signaling and glutamate signaling. Elevated expression of LGMN in human white matter active and chronic active lesions was associated with activated MHC class II positive microglia and macrophages, which could have clinical applicability in detecting ongoing inflammatory activity in multiple sclerosis if a corresponding association could be verified in serum or CSF of MS patients. The proteins LGMN, HEMO and C1Q were not significantly altered in CSF from multiple sclerosis patients investigated. Even if the number of patients in each category is small, this suggests that measurements of those proteins in CSF will have limited value in separating the examined groups.

## Supplementary Information

Supplementary Figure S1.

Supplementary Figure S2.

Supplementary Information.

Supplementary Dataset 1.

Supplementary Dataset 2.

Supplementary Dataset 3.

Supplementary Legends.

## Abbreviations

CNS: Central nervous system
CPZ: Cuprizone
CTR: Control
EAE: Experimental autoimmune encephalomyelitis
EDSS: Expanded disability status scale
GFAP: Glial fibrillary acidic protein
HE: Hematoxylin–eosin
HLA-DR: Human leukocyte antigen-DR
HPLC: High-pressure liquid chromatography
IPA: Ingenuity pathway analysis
LC–MS: Liquid chromatography–mass spectrometry
LFB: Luxol fast blue
LGMN: Legumain
Mac-3: Lysosomal-associated membrane protein 2
MHC: Major histocompatibility complex
NOGO-A: Neurite outgrowth inhibitor protein A
OND: Other neurological disease
PLP: Proteolipid protein
PRM: Parallel reaction monitoring
RT: Room temperature
Ser: Serine
SPSS: Statistical package for social sciences
STAT: Signal transducer and activator of transcription
TCEP: Tris(2-carboxyethyl)phosphine
TEAB: Tetraethylammonium bromide
Thr: Threonine
TMT: Tandem mass tag
Tyr: Tyrosine
