# Supplementary figures and images for: Cuprizone and EAE mouse frontal cortex proteomics revealed proteins altered in multiple sclerosis
Source: Sci Rep. 2021 Mar 30;11:7174. doi: 10.1038/s41598-021-86191-5 (PMC8010076; doi:10.1038/s41598-021-86191-5)

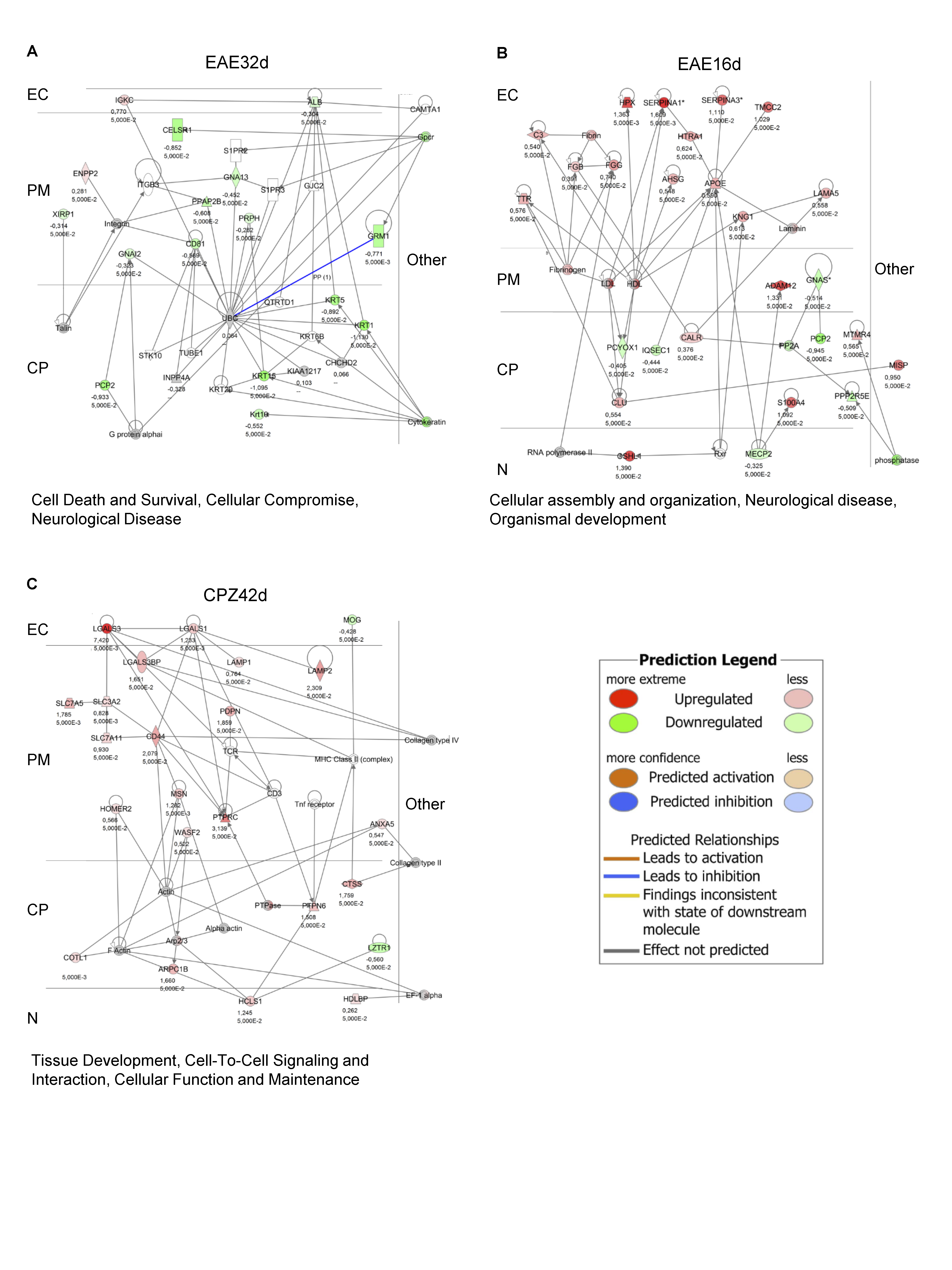

Supplement: Supplementary file 2 — Supplementary Figure S2. [file 41598_2021_86191_MOESM2_ESM.tif]
