# Supplementary material for: Cuprizone and EAE mouse frontal cortex proteomics revealed proteins altered in multiple sclerosis
Source: Sci Rep. 2021 Mar 30;11:7174. doi: 10.1038/s41598-021-86191-5 (PMC8010076; doi:10.1038/s41598-021-86191-5)
Supplement: Supplementary file 3 — Supplementary Information. [file 41598_2021_86191_MOESM3_ESM.docx]

Supplementary methods

Cuprizone and EAE mouse frontal cortex proteomics revealed proteins altered in multiple sclerosis

Eystein Oveland^1^, Intakhar Ahmad^2,3^, Ragnhild Reehorst Lereim^1,2^, Ann Cathrine Kroksveen^1,2^, Harald Barsnes^1,4^, Astrid Guldbrandsen^1,~~2~~,3^, Kjell-Morten Myhr^2,6,^ , Lars Bø^2,3,6^, Frode S. Berven^1,3*^, Stig Wergeland ^3,5,6*§^

^1^ Proteomics Unit at the University of Bergen (PROBE), Department of Biomedicine, University of Bergen, Norway.

^2^ Department of Clinical Medicine, University of Bergen, Norway.

^3^ Norwegian Multiple Sclerosis Competence Centre, Department of Neurology, Haukeland University Hospital, Bergen, Norway.

^4^ Department of Clinical Science, University of Bergen, Norway.

^5^ The Norwegian Multiple Sclerosis Registry and Biobank, Department of Neurology, Haukeland University Hospital, Bergen, Norway.

^6^ Neuro-SysMed, Department of Neurology, Haukeland University Hospital, Bergen, Norway.

^*^Co-senior authors

# Immunohistochemistry

Specification of antibodies and staining protocols

| Antibody | Provider | Reactivity | Dilution | Antigen retrieval | Incubation |
| --- | --- | --- | --- | --- | --- |
| Proteolipid protein (PLP) | Serotec | Mouse | 1:1000 |  | ON @ 4 ℃ |
| Neurite outgrowth inhibitor protein A (NOGO-A) | Chemicon | Mouse | 1:1000 |  | 1h @ RT |
| Mac-3 | BD Biosciences | Mouse | 1:200 |  | ON @ 4 ℃ |
| Legumain (LGMN) | Sigma-Aldrich | Human | 1:200 | DIVA | 2hrs @ RT |
| PLP | Abcam | Human | 1:1000 | DIVA | ON @ 4 ℃ |
| HLA-DR | DAKO (Agilent) | Human | 1:20 | DIVA | 2hrs @ RT |
| GFAP | DAKO (Agilent) | Human | 1:50 | DIVA | 2hrs @ RT |
| O1 | Thermo Fisher Scientific Inc. | Human | 1:50 | DIVA | 2hrs @ RT |
| O4 | MyBioSource | Human | 1:50 | DIVA | 2hrs @ RT |
| ON: Overnight, RT: Room temperature | | | | | |

# Digitalization of stained sections

PLP, HLA and LGMN stained sections were digitized using a Scanscope XT slide scanner (Aperio Technologies; Vista, CA) using an Olympus UPLSAPO 40× objective (Olympus, Southend-on-Sea, UK) and Techniquip Model 21DC Light source (Techniquip, Pleasanton, California, USA), with a GE quartzline projector lamp model EKE 21 V 150 W (General Electric, Fairfield, Connecticut, USA). Images were scanned with a final resolution of 0.247 μm per pixel. The virtual slides were saved using the Aperio NDPI file format. Virtual slides were viewed in Aperio Imagescope software V.12 (Aperio, Vista, California, USA).

# Experimental autoimmune encephalomyelitis

25 µg recombinant human MOG (rh-MOG, MOG1-125 from Hooke Labs, Lawrence, MA) was emulsified in Freund’s adjuvant with 1mg/ml M. tuberculosis H37RA (Sigma-Aldrich) to a total volume of 0.1ml and injected subcutaneously at Day 0 (day of immunization). In addition, 200 ng of Pertussis toxin (Sigma-Aldrich) was injected intra-peritoneally at Day 0 and at post-immunization (p.i.) Day 2. The EAE clinical disease activity was scored from 0-8 (0: Healthy, 1: Tail weakness, 2: Tail paralysis, 3: Mild hind leg paresis, 4: Severe hind leg paresis, 5: Paralysis of one hind leg, 6: Complete paralysis of both hind legs, 7: Tetraparalysis, 8: Death) ([Supplementary Fig. 1](SFile01_Figure%20S1.tif)).

Tissue homogenization and protein extraction

The brain tissue was thawed on ice and immediately added 200 µl 50 mM triethylammonium bicarbonate (TEAB) buffer containing 8 M urea, 30 μl/ml complete mini protease inhibitor cocktail (Roche), 1 mM heat-activated sodium vanadate (Tyr phosphatase inhibition) and 10 mM NaF (Ser/Thr-phosphatase inhibition). The tissue was then homogenized by sonication (High Intensity Ultrasonic Processor 50 W, Sonics & Materials Inc.). The sample was left on ice for 30 min, centrifuged at 16.000g for 20 min, and the supernatant was transferred to a fresh tube and stored at -80ºC until LC-MS/MS analyses.

# TMT-labeling and protein quantification

## Mixed mode fractionation of the TMT-labeled peptides sample

The TMT-labeled tryptic peptides were fractionated in 60 fractions using mixed-mode HPLC chromatography. The dried TMT-labeled peptides (TMT-mix) were reconstituted in 400 µl buffer A (20 mM ammonium formate, 3% acetonitrile (ACN)). 100 µl of the sample was loaded onto a Sielc Promix column (MP-10.250.0530, 1.0 × 250 mm, 5 μm, 300Å, Sielc Technologies) using an Agilent 1260 LC system with Chemstation Rev. B0.4.0 (Agilent Technologies) using 85% A for 10 min at a flow rate of 50 μl/min. The peptides were eluted using a gradient of 15% - 60% buffer B (2 mM ammonium formate, 80% ACN, pH 3.0) over 35 min, 60%-100% B over 10 min and held constant for 5 min. The sample was fractionated into 60 fractions in a 96-well plate, one fraction (approximately 58.5 µl) was collected every 1.16 min until 70 min. The plate was frozen in -80ºC and peptides lyophilized in a vacuum concentrator (Centrivap with a Cold trap, Labconco) at 30ºC. The peptide powder was reconstituted in 10 μl 0.1% FA and transferred to HPLC-tubes.

## Orbitrap LC-MS of TMT-labeled mixed mode fractions

Approximately 0.5 μg of TMT-labeled peptides were loaded and desalted on a pre-column (Acclaim PepMap 100, 2cm x 75µm i.d. nanoViper column, packed with 3 µm C18 beads, Thermo Scientific). The flow rate was 5 µl/min for 6 min with an isocratic flow of 0.1 % FA with 2% ACN using an Ultimate NCS-3500RS (Dionex). Peptide separation and elution were accomplished on an analytical column (Acclaim PepMap 100, 15 cm x 75 µm i.d. nanoViper column, packed with 2 µm C18 beads) using a biphasic ACN gradient from two nanoflow UPLC pumps (flow rate of 280 nl/min). Solvent A was 0.1% FA with 2% ACN and solvent B 0.1% FA with 90% ACN. The HPLC gradient was generated by mixing solvent A with the following percentage of solvent B: 5% from 0 to 5 min, up to 8% from 5 to 6 min, up to 20% from 6 to 60 min, up to 35% from 60 to 90 min, up to 90% from 90 to 100 min, held at 90% from 100 to 105 min, down to 5% from 105 to 108 min, and held at 5% until 120 min.

The eluting peptides were ionized in the electrospray and analyzed by the LTQ-Orbitrap Velos Pro (Thermo Scientific). The mass spectrometer was operated in the DDA-mode (data-dependent-acquisition) to automatically switch between one survey full scan MS (FTMS) and 14 MS/MS acquisitions (ITMS). The instrument control was through Tune 2.7 and Xcalibur 2.2. The FTMS scans (from 300 to 2000 m/z) were acquired for 110 min in the Orbitrap with a resolution R = 60000 at 400 m/z (after accumulation to a target value of 1E6 in the linear ion trap with maximum allowed ion accumulation time of 500 ms).

The 7 most intense eluting peptides above an ion threshold value of 1000 counts and charge states 2 or higher, were sequentially isolated in a back-to-back analysis of same-precursors using two different fragmentation techniques, (i) CID (Collision-Induced Dissociation) and (ii) HCD (Higher-Energy Collision Dissociation).

(i) Ions were isolated to a target value of 1E4 at a maximum ion accumulation time of 200 ms, and fragmented in the high-pressure linear ion trap by low-energy CID with normalized collision energy of 35% and wideband-activation enabled. The maximum allowed accumulation time for CID was 200 ms, isolation width maintained at 2 Da, activation q = 0.25, and activation time of 10 ms. Fragments were detected in the low-pressure ion trap at normal scan rate, and recorded with the secondary electron multipliers.

(ii) Ions were isolated in the high-pressure linear ion trap to a target value of 5E5 at a maximum allowed accumulation time of 1000 ms, and isolation width maintained at 3 Da. Fragmentation in the HCD cell was performed with a normalized collision energy of 40%, and activation time of 0.1 ms. Fragments were detected in the Orbitrap at a resolution of 7500 with first mass fixed at 90 m/z.

Two MS/MS spectra of a precursor mass were allowed before dynamic exclusion for 20 s. Lock-mass internal calibration was not enabled.

## Analysis of the TMT LC-MS data using Proteome Discoverer

Proteome Discoverer version 1.4.1.14 (Thermo Scientific) was used for quantification and identification of the TMT data. MS Amanda and SEQUEST were used as search engines and the Swissprot database *Mus musculus* (with canonical sequence data in FASTA version 27.03.14) was used. The enzyme was set to trypsin and the max missed cleavages to 2. The precursor mass tolerance was 10 ppm with accepted precursor charges from +2 to +7, and the fragment mass tolerance was 0.05 Da. Carbamidomethyl of C, TMT6plex on K and TMT6plex on any N-terminus were set to fixed modifications. Oxidation of M was set to variable modification. The perculator was used with target FDR strict 0.01 and relaxed 0.05. The method “Most Confident Centroid” without Quan Value Corrections (recommended in the PD v1.4 manual) and an integration tolerance of 1000 ppm were used for quantification of the TMT peaks in the HCD spectra.

# Label-free protein quantification

## Protein digestion and peptide clean-up

Based on the protein measurements a volume corresponding to 10 μg of protein was denatured by adding 20 μl of Urea solution (8M urea/20 mM methylamine). The sample was then added 20 μl of Trypsination buffer (50 mM Tris HCl pH 8.0/1 mM CaCl_2_) and shaken at RT for 5 min at 300 rpm in an Eppendorf mixer (Eppendorf, Hamburg, Germany).

Proteins were reduced by adding 4 μl of fresh 100 mM DTT to the tube and incubated for 1 hour at RT. The proteins were then alkylated by adding 5 μl of fresh 200 mM iodoacetamide and incubating for 1 h at RT in the dark. A volume of 0.8 μl 100 mM DTT was added and the sample incubated for 10 min.

A volume of 110 μl of Trypsination buffer was added to reduce the urea concentration to 1 M. Trypsin from a vial (Trypsin porcine, Promega art. No. V 5111) dissolved in 50 mM acetic acid was added to obtain a trypsin:protein ratio of 1:50 (w/w). The sample was incubated in an Eppendorf shaker at 300 rpm for 16h at 37ºC. After 16 h the trypsination was stopped by adding 15 μl of 10 % formic acid (FA).

The tryptic peptide mixture was desalted and concentrated using Oasis HLB uElution Plate 30 um (Part no 186001828BA, Waters). The eluted peptides were frozen in -80ºC and lyophilized in a vacuum concentrator (Centrivap with a Cold trap, Labconco) set to 30ºC. The peptides were reconstituted in 1 μl 100% FA and diluted to 0.5 μg/μl and 5% FA by adding 19 μl H_2_O.

## Orbitrap LC-MS of label-free samples

Tryptic peptides (2.5 µg) were separated during a biphasic acetonitrile (ACN) gradient from two nanoflow UPLC pumps (flow rate of 280 nl/min) on a 50 cm analytical column (Dionex #164570, Acclaim PepMap100 nanoViper column, 75 μm i.d. × 50 cm, packed with 3 μm C18 beads). Solvent A was 0.1% FA (vol/vol) with 2% ACN and solvent B was 0.1% FA (vol/vol) with 90% ACN (vol/vol). The gradient composition was solvent A for 5 minutes, then from 5 to 8% solvent B over 0.5 min, then 8% B for 4.5 min, then 8 to 25% B over 100 min, then 25 to 45% B over 60 min, then 45 to 90% B over 10 min, then 90% B for 10 min, followed by 90 to 5% B over 3 min, and finally 5% solvent B for 27 min. The gradient time (from 5 to 90% solution B) and the acquisition time for MS data were 180 min, and the whole HPLC run was 220 min.

The peptide samples (2.5 µg) were subjected to LC-MS analysis using 220 min runs with a biphasic acetonitrile gradient and a 50 cm nanoViper column using an Ultimate NCS-3500RS HPLC coupled to an LTQ-Orbitrap Velos Pro. The eluting peptides were ionized in the electrospray and analyzed by an LTQ-Orbitrap Velos Pro. The mass spectrometer was operated in the DDA-mode (data-dependent-acquisition) to automatically switch between full scan MS and MS/MS acquisition. Instrument control was through Tune 2.7 and Xcalibur 2.2. Survey full scan MS spectra (from 300 to 2000 m/z ) were acquired for 180 min in the Orbitrap with a resolution R = 60000 at 400 m/z (after accumulation to a target value of 1E6 in the linear ion trap with maximum allowed ion accumulation time of 500 ms). The 7 most intense eluting peptides above a ion threshold value of 1000 counts, and charge states 2 or higher, were sequentially isolated to a target value of 1E4 and fragmented in the high-pressure linear ion trap by low-energy CID (collision-induced-dissociation) with normalized collision energy of 40% and wideband-activation enabled. The maximum allowed accumulation time for CID was 200 ms, the isolation width maintained at 2 Da, activation q = 0.25, and activation time of 10 ms. The resulting fragment ions were scanned out in the low-pressure ion trap at normal scan rate, and recorded with the secondary electron multipliers. One MS/MS spectrum of a precursor mass was allowed before dynamic exclusion for 20 s. Lock-mass internal calibration was not enabled.

## Analysis of the label-free LC-MS data using Progenesis LC-MS

Progenesis LC-MS® v2.6 (Nonlinear Dynamics Ltd) was used for LF quantification and comparison of LC-MS proteomics data based on the volume, m/z and retention time of the MS^1^ features (peptides). In Progenesis, the LC-MS runs were automatically aligned, and only features with charges between +2 to +7 and containing associated MS/MS spectra were accepted for export as an mgf file for identification. The mgf file was searched against the UniProt database *Mus musculus* (SwissProt with canonical sequence data in FASTA version September 2012) using SearchGUI v1.8.9 ([Vaudel *et al.*, 2011](#_ENREF_1)) . The search criteria were: trypsin as the protease with one miss-cleavages accepted, fixed carbamidomethylation on cystein, variable oxidation on methionine, precursor mass tolerance of 10 ppm, fragment mass tolerance of 0.7 and X!Tandem (version Cyclone) as the search engine. The search result and associated spectra were combined and assigned to proteins in PeptideShaker v0.17.3 ([Vaudel *et al.*, 2015](#_ENREF_2)) at 1% FDR. The results were exported from PeptideShaker as validated PSMs in a Phenyx format, and imported back into Progenesis. The protein abundances reported from Progenesis were based on the sum of the normalized abundance of the identified unique peptides.

# Parallel reaction monitoring (PRM) targeted quantification

# Protein concentration in the CSF pools was measured by the BCA (Pierce), following the manufacturers protocol. All CSF samples were in-solution digested as previously described (4). About 1.5 µg CSF digested protein were injected for Legumain and C1q and about 0.2 ug for Hemopexin.

# *Preparation and spike-in of synthetic peptides*

# All isotopic labelled peptides (IS peptides), C-terminally modified with 13C and 15N isotope arginine or lysine, used as internal standards were purchased from JPT Peptide Technologies with the highest quality (>95% purity) quality. The amount of spike-in per ug of CSF digested protein was 3 fmol for LGMN, 100 for HEMO and 14 fmol for C1Q . 2+ versions of the peptides were monitored.

# *PRM mass spectrometry*

# The separation of peptides was performed by an Ultimate™ 3000 RSLCnano System (Thermo Fisher Scientific™) with an Acclaim PepMap™ 100 trap column (diameter width at 75 µm x 2 cm nanoviper C18 column, with particle size 3 µm and length at 100 A) and 5 µL 0.1 % TFA solution. Peptides were separated on an analytical column PepMapTM RSLC C18 ES802 (diameter width 75 µm x 25 cm length, particle size at 2 µm) For the 90 minute method, the LC gradient was a combination of 95 % solvent A (0.1 % FA) and 5 % solvent B (100 % ACN, 0.1 % FA) with a flow rate of 250 µl/min. The column gradient for peptide elution went from 0-5.5 min with 5 % solvent B, then an increase at 5.5– 7 min to 7 % of solvent B, 7 – 50 min increase to 22 % B, 50 – 65 min increase to 35 % B and 65 – 70 min increase to 80 % B. At 70 – 77 min solvent B was kept constant at 80% B, from 77-80 min decreased to 5 % B and held at 5 % solvent B from 80 – 90 min.

# For the 40 minute method, the LC gradient was a combination of 95 % solvent A (0.1 % FA) and 5 % solvent B (100 % ACN, 0.1 % FA) with a flow rate of 250 µl/min. The column gradient for peptide elution went from 0-5.5 min with 5 % solvent B, then an increase at 5.5 – 7 min to 7 % of solvent B, 7 – 16.5 min increase to 25 % B, 16.5 – 22.2 min increase to 40 % B and 22.2 – 22.6 min increase to 80 % B. At 22.6 – 29.2 min solvent B was kept constant at 80% B, from 29.2-31 min decreased to 5 % B and held at 5 % solvent B from 31 – 40 min.

# The mass spectrometry method duration was 90 min (for LGMN and C1q) and 40 min (for HEMO). The Q-Exactive HF mass spectrometer controlled through Q Exactive HF Tune 2.4 and Xcalibur 3.0 was operated in PRM scheduled mode with on full scan MS1 between every 12th PRM MS2 scan. The target peptides on the inclusion list were sequentially isolated for higher-energy collision dissociation (HCD) fragmentation and MS2 acquisition resolution was 60 000, automatic gain control (AGC) target of 2e5, isolation window 0.7m/z, CE 28 and a maximum injection time (IT) of 120 ms (118 ms for the 90 min method). Lock-mass (445.12003 m/z) internal calibration was used.

# *Skyline analysis*

# Skyline (29) settings were overall kept at default. Notably, structural modifications were specified with carbamidomethyl (C) and isotope modification “label: 13C(6) 15N(2) (C-term K)” and label: “13C(6) 15N(4) (C-term R)”.

# The peak signal for each peptide was determined by the Skyline peak picking algorithm, and manually verified or re-integrated based on the fragment pattern of the peptide, elution profile and simultaneous retention time of the endogenous and the IS peptide. The three fragments with the highest intensity, low interference, and mass error less than 10 ppm was selected for quantitation. The area under the curve, excluding background, were summed to give one peak area value for each peptide.

# Furthermore, the endogenous peak area was divided by the peak area of the heavy internal standard peptide to generate a ratio to standard used for calculating the amount of endogenous peptide in each sample in fmol/ug. An unpaired two tailed student’s t-test was used to compare disease groups and a p-value of ≤ 0.05 would indicate significant difference.

Post-processing of proteomics data and availability

Perseus v1.4.1.3 was used to generate the unsupervised clustering heatmap with dendrograms of z-score normalized data using the default settings. GraphPad Prism 6 (GraphPad Prism Software) was used for the statistical analyses and graphics and Venn diagrams were created using Venny (http://bioinfogp.cnb.csic.es/tools/venny/index.html).

Ingenuity Pathway Analysis (IPA, Ingenuity Systems, http://www.ingenuity.com) was used for pathway and function analyses. The protein lists from the TMT-labelling and label-free experiments were combined and the average of the quantification values were used for the proteins detected in both experiments. In the IPA analysis, the proteins significantly altered with a fold change higher than 20% in both TMT and label-free were considered more significant (IPA P-value 0.005) than the proteins significantly regulated in only one of the experiments (IPA p-value 0.05). The relationships were set to “experimentally observed direct interactions”. IPA reported the likelihood that proteins were associated with a pathway or upstream regulator (p<0.05 = significant) and the predicted direction of the change (z ≤ 2 = decreased; z ≥ 2 = increased). DAVID [24] was used to investigate the regulated proteins in KEGG pathways [25].

# MADGENE [26] was used to convert the mouse accession numbers to human orthologues for comparison against the results in CSF-PR [14]. The Basic Local Alignment Search Tool (BLAST) in UniProt was used with default settings for the accession numbers not found by MADGENE.

# References

Vaudel M, Barsnes H, Berven FS, Sickmann A, Martens L. SearchGUI: An open-source graphical user interface for simultaneous OMSSA and X!Tandem searches. Proteomics 2011; 11: 996-9.

Vaudel M, Burkhart JM, Zahedi RP, Oveland E, Berven FS, Sickmann A, et al. PeptideShaker enables reanalysis of MS-derived proteomics data sets. Nat Biotechnol 2015; 33: 22-4.
