# Supplementary material for: Cuprizone and EAE mouse frontal cortex proteomics revealed proteins altered in multiple sclerosis
Source: Sci Rep. 2021 Mar 30;11:7174. doi: 10.1038/s41598-021-86191-5 (PMC8010076; doi:10.1038/s41598-021-86191-5)
Supplement: Supplementary file 7 — Supplementary Legends. [file 41598_2021_86191_MOESM7_ESM.docx]

# Supplementary material

Supplementary File 1: Supplementary Figure 1. EAE and CPZ mice disease course and histopathology.

The average weight of the (**A)** CPZ mice and (**B**) EAE mice were lower than for the respective controls as previously reported (Wergeland *et al.*, 2011). (**C**) For CPZ mice (sacrificed at day 42) we observed demyelination (luxol fast blue, Anti-PLP), increased number of activated microglia cells (anti-Mac3) and a loss of oligodendrocytes (anti-NOGO-A) in accordance with previous results (Wergeland *et al.*, 2012a). (**D**) The clinical score of the EAE mice at different time points after peritoneal injection of rhMOG1-125, the scoring of the mice sacrificed after 16d (EAE-16d, disease peak) and after 32d (EAE-32d, partial recovery) are shown (insert); the respective control animals had a clinical score of zero. Error bars represent +/- 1 SD.

Supplementary File 2: Supplementary Figure 2. Predicted protein networks and diseases from EAE and CPZ proteomics data.

The protein ratios relative to respective controls for EAE-16d, EAE-32d and CPZ-42d from TMT and label-free were combined prior to analyses in IPA. The significance level of regulation (IPA “p-value”) was set to 0.005 if the protein was significantly regulated with more than 1.2-fold in both TMT and label-free experiments and 0.05 if in only one experiment. The average log_2_ ratio and the IPA “p-value” from the combined TMT and label-free protein list are shown for each protein node. Networks predicted with high score in IPA are shown for (**A**) EAE-32d, (**B**) EAE-16d and (**C**) CPZ-42d.

Supplementary File 3. Supplementary methods.

Supplementary File 4. Supplementary Data 1. All proteins quantified using TMT including label free if found.

Supplementary File 5. Supplementary Data 2. All proteins quantified using label-free proteomics.

Supplementary File 6. Supplementary Data 3. PRM quantification of LGMN, C1Q and HEMO in human CSF.
